# On strongly primary monoids and domains

**DOI:** 10.1080/00927872.2020.1755678

**Published:** 2020-05-04

**Authors:** Alfred Geroldinger, Moshe Roitman

**Affiliations:** a Institute of Mathematics and Scientific Computing, University of Graz, NAWI Graz, Graz, Austria;; b Department of Mathematics, University of Haifa, Haifa, Israel

**Keywords:** Local tameness, one-dimensional local domains, primary monoids, sets of distances, sets of lengths, 13A05, 13F05, 20M13

## Abstract

A commutative integral domain is primary if and only if it is one-dimensional and local. A domain is strongly primary if and only if it is local and each nonzero principal ideal contains a power of the maximal ideal. Hence, one-dimensional local Mori domains are strongly primary. We prove among other results that if R is a domain such that the conductor $\left(R:\hat{R}\right)$ vanishes, then Λ(R) is finite; that is, there exists a positive integer k such that each nonzero nonunit of R is a product of at most k irreducible elements. Using this result, we obtain that every strongly primary domain is locally tame, and that a domain R is globally tame if and only if Λ(R)=∞. In particular, we answer Problem 38 of the open problem list by Cahen et al. in the affirmative. Many of our results are formulated for monoids.

## 1. Introduction

Factorization theory of integral domains studies factorizations of elements and ideals ([1, 11, 18]). Some ideal theoretic conditions (such as the ascending chain condition on principal ideals) guarantee that every nonzero, nonunit element of a domain can be written as a product of atoms (irreducible elements). The goal is to describe the nonuniqueness of factorizations by arithmetical invariants and study their relationship with classical ring theoretical invariants. Class groups and the structure of certain localizations are such algebraic invariants which control element factorizations. In case of weakly Krull Mori domains, this interplay is described by the *T*-block monoid of the domain which is built by the *v*-class group and localizations at height-one primes containing the conductor ([18, Theorem 3.7.1]). These localizations are one-dimensional and local, and this connection stimulated the interest of factorization theory in one-dimensional local domains.

To recall some arithmetical invariants, let *R* be an integral domain. If $a=u_{1}\cdot \ldots \cdot u_{k}$ is a factorization of an element a∈R into atoms $u_{1},\ldots ,u_{k},$ then *k* is a factorization length. The set L(a)⊆N of all possible factorization lengths of *a* is called the *set of lengths* of *a*. The *local tame degree* t(u) of an atom u∈R is the smallest *N* with the following property: For any multiple *a* of *u* and any factorization $a=v_{1}\cdot \ldots \cdot v_{n}$ of *a*, there is a subproduct which is a multiple of *u*, say $v_{1}\cdot \ldots \cdot v_{m},$ and a refactorization of this subproduct which contains *u*, say $v_{1}\cdot \ldots \cdot v_{m}=uu_{2}\cdot \ldots \cdot u_{\ell }$ such that max{ℓ,m}≤N. Local tameness is a central finiteness property which – in many settings – implies further arithmetical finiteness properties (see, e.g. [16, 18, Section 4]).

Here are two classes of locally tame monoids:

- Krull monoids with finite class group [18, Theorem 3.4.10];
- C-monoids [18, Theorem 3.3.4].

Moreover, if *R* is a Mori domain with nonzero conductor $f=(R:\hat{R})$ such that R/f and the class group $C(\hat{R})$ are finite, then the multiplicative monoid $R^{•}=R\setminus \{0\}$ is a C-monoid by [18, Theorem 2.11.9], whence *R* is locally tame (compare with Example 3.17.4). Precise values for local and global tame degrees were studied for Krull monoids and numerical monoids ([8, 9, 12, 31]). For computational aspects and for results in the noncommutative setting we refer to [5, 13].

By a *local* ring, we mean a commutative ring with a unique maximal ideal, not necessarily Noetherian. It is well known that a domain *R* is one-dimensional and local if and only if its multiplicative monoid of nonzero elements is primary. A monoid *H* is *strongly primary* if each principal ideal of *H* contains a power of the maximal ideal. The multiplicative monoid of a one-dimensional local Mori domain is strongly primary, and this was the key property to prove local tameness for one-dimensional local Mori domains under a variety of additional assumptions ([22, Theorem 3.5] and [27, Theorem 3.5]). However, the general case remained open.

In the present paper, we prove that every strongly primary domain is locally tame and we provide a characterization of global tameness (Theorems 3.8 (b) and 3.^9^.^2^). In particular, all one-dimensional local Mori domains turn out to be locally tame and this answers Problem 38 in [7] in the affirmative. Although our present approach is semigroup theoretical over large parts (Theorem 3.8 (a)), it also uses substantially the ring structure, and this is unavoidable since strongly primary Mori monoids need not be locally tame as shown in [22, Proposition 3.7 and Example 3.8] (see Example 3.17.1).

## 2. Background on primary monoids and domains

We denote by N the set of positive integers and by $N_{0}=N\cup \{0\}$ the set of non-negative integers. For integers a,b∈Z, we denote by [a,b]={x∈Z|a≤x≤b} the discrete interval between *a* and *b*. Let L,L′⊆Z be subsets of the integers. Then L+L′={a+b|a∈L,b∈L′} denotes the sumset. A positive integer d∈N is a distance of *L* if there is some a∈L such that L∩[a,a+d]={a,a+d}. We denote by Δ(L)⊆N the set of distances of *L*. Thus, Δ(L)=∅ if and only if |L|≤1, and *L* is an arithmetical progression with difference *d* if and only if Δ(L)⊆{d}. Note that the empty set is considered an arithmetical progression.

By a *monoid*, we mean a commutative cancellative semigroup with identity and by a *domain*, we mean a commutative integral domain. Thus, if *R* is a domain, then $R^{•}=R\setminus \{0\}$ is a monoid. All ideal theoretic and arithmetical concepts are introduced in a monoid setting, but they will be used both for monoids and domains.

**Ideal theory of monoids and domains.** Let *H* be a monoid and q(H) the quotient group of *H*. We denote by

- $H\prime =\{x\in q(H)|\text{there}\;\text{exists}\;\text{some}\;N\in N\;\text{such}\;\text{that}\;x^{n}\in H\;\text{for}\;\text{all}\;n\geq N\}$ the *seminormal closure* of *H*, by
- $\tilde{H}=\{x\in q(H)|\text{there}\;\text{exists}\;\text{some}\;N\in N\;\text{such}\;\text{that}\;x^{N}\in H\}$ the *root closure* of *H*, and by
- $\hat{H}=\{x\in q(H)|\text{there}\;\text{exists}\;c\in H\;\text{such}\;\text{that}\;cx^{n}\in H\;\text{for}\;\text{all}\;n\in N\}$ the *complete integral closure* of *H*.

Then, we have

(2.1) $$H\subseteq H\prime \subseteq \tilde{H}\subseteq \hat{H}\subseteq q(H)\;,$$

and all inclusions can be strict. We say that *H* is *seminormal* (*root-closed*, resp. *completely integrally closed*) if H=H′ ($H=\tilde{H},$ resp. $H=\hat{H}$). Note that H′ is seminormal, $\tilde{H}$ is root-closed, but $\hat{H}$ need not be completely integrally closed.

Lemma 2.1. *Let H be a root-closed monoid and* x∈q(H).

*1. If* c∈H *such that* $cx^{n}\in H$ *for some* n∈N*, then* $cx^{k}\in H$ *for every* k∈[1,n].

*2. We have* $x\in \hat{H}$ *if and only if there exists* c∈H *such that* $cx^{n}\in H$ *for infinitely many* n∈N.

Proof. 1. Let c∈H and n∈N such that $cx^{n}\in H.$ If k∈[1,n], then ${\left(cx^{k}\right)}^{n}={\left(cx^{n}\right)}^{k}c^{n-k}\in H$ whence $cx^{k}\in H$ because *H* is root-closed.

2. If there is a c∈H such that $cx^{n}\in H$ for infinitely many n∈N, then 1. implies that $cx^{k}\in H$ for all k∈N whence $x\in \hat{H}.$ □

Lemma 2.2. Let H be a monoid.

1. $\hat{H}$ *is root-closed.*

*2. If* $(H:\hat{H})\neq \emptyset$*, then* $\hat{H}$ *is completely integrally closed.*

*3. An element* x∈q(H) *lies in* $\hat{\hat{H}}$ *if and only if there exists an element* c∈H *such that* $cx^{n}\in \hat{H}$ *for infinitely many* n∈N.

Proof. 1. Let x∈q(H) such that $x^{e}\in \hat{H}$ for some e∈N. We have to show that $x\in \hat{H}.$ There is an element c∈H such that $cx^{i}\in H$ for every i∈[1,e] and such that $c{\left(x^{e}\right)}^{k}\in H$ for all $k\in N_{0}.$ If n∈N, then n=ke+i, with $k\in N_{0}$ and i∈[1,e], and $c^{2}x^{n}=c{\left(x^{e}\right)}^{k}(cx^{i})\in H$ which implies that $x\in \hat{H}.$

2. See [18, Proposition 2.^3^.^4^].

3. Since $\hat{H}$ is root-closed by 1., the assertion follows from Lemma 2.1.2 (applied to the monoid $\hat{H}$). □

Let *H* be a monoid. Then, $H^{\times }$ denotes the group of units of *H* and $H_{\text{red}}=\{aH^{\times }|a\in H\}$ its associated reduced monoid. We also let $m=H\setminus H^{\times }$ and $n=\hat{H}\setminus {\hat{H}}^{\times }.$ Let X,Y⊆q(H) be subsets. Then, *X* is an *s*-ideal of *H* if X⊆H and *XH* = *X*. We set (X:Y)={z∈q(H)|zY⊆X} and $X^{-1}=(H:X).$ A *divisorial ideal (v-ideal)* is a set of the form ${\left(X^{-1}\right)}^{-1}$ for X⊆q(H). We denote by *v*-spec(H) the set of all prime *v*-ideals of *H*. The monoid *H* is a *Mori monoid* if it satisfies the ascending chain condition on divisorial ideals, and it is a *Krull monoid* if it is a completely integrally closed Mori monoid.

**Arithmetic of monoids and domains.** For any set *P*, let F(P) be the free abelian monoid with basis *P*. Let $|\cdot |:F(P)\to N_{0}$ denote the unique epimorphism satisfying |p|=1 for each p∈P, whence |·| is mapping each z∈F(P) onto its length. We denote by A(H) the set of atoms of *H*. Then, $Z(H)=F(A(H_{\text{red}}))$ is the *factorization monoid* of *H* and $\pi :Z(H)\to H_{\text{red}}$ denotes the canonical epimorphism. For an element a∈H,

- $Z(a)=\pi^{-1}(aH^{\times })\subseteq Z(H)$ is the *set of factorizations* of *a*, and
- $L(a)=\{|z||z\in Z(a)\}\subseteq N_{0}$ is the *set of lengths* of *a*.

To define the distance of factorizations, consider two factorizations z,z′∈Z(H). Then, we write

$$z=u_{1}\cdot \ldots \cdot u_{\ell }v_{1}\cdot \ldots \cdot v_{m}\;\text{and}\;z\prime =u_{1}\cdot \ldots \cdot u_{\ell }w_{1}\cdot \ldots \cdot w_{n}\;,$$

where $\ell ,m,n\in N_{0}$ and all $u_{i},v_{j},w_{k}\in A(H_{\text{red}})$ such that $\{v_{1},\ldots ,v_{m}\}\cap \{w_{1},\ldots ,w_{n}\}=\emptyset .$ We call $d(z,z\prime )=\max\{m,n\}\in N_{0}$ the *distance* between *z* and z′. The function $d:Z(H)\times Z(H)\to N_{0}$ is a metric on Z(H). We say that *H* is

- *atomic* if every nonunit can be written as a finite product of atoms, and
- a BF-*monoid* (a *bounded factorization monoid*) if it is atomic and all sets of lengths are finite.

A monoid is a BF-monoid if and only if $\underset{n\geq 0}{\cap }{\left(H\setminus H^{\times }\right)}^{n}=\emptyset ,$ and Mori monoids are BF-monoids ([18, Theorem 2.2.9]). For every k∈N, we set $\rho_{k}(H)=k$ if $H=H^{\times },$ and otherwise we set

$$\rho_{k}(H)=\sup\{\sup L(a)|a\in H,k\in L(a)\}\in N\cup \{\infty \}\;.$$

Clearly, the sequence ${\left(\rho_{k}(H)\right)}_{k\geq 1}$ is increasing and, if $\rho_{k}(H)$ is finite for some k∈N, then $\rho_{k}(H)$ is the maximal length of a factorization of a product of *k* atoms. The *elasticity* of *H*, introduced by Valenza in [34], is defined as ρ(H)=sup{m/n|m,n∈L,L∈L(H)}, and by [18, Proposition 1.4.2] we have

(2.2) $$\rho (H)=\sup\{m/n|m,n\in L,L\in L(H)\}=\underset{k\to \infty }{\lim}\frac{\rho_{k}(H)}{k}.$$

For a subset S⊆H, the *set of distances* of *S* is defined by

$$\Delta (S)=\underset{a\in S}{\cup }\Delta (L(a))\;\text{and}\;\text{we}\;\text{set}\;\Lambda (S)=\sup\{\min L(a)|a\in S\}\in N_{0}\cup \{\infty \}\;.$$

For a∈H, let Λ(a)=Λ({a}). If a,b∈H, then L(a)+L(b)⊆L(ab) whence minL(ab)≤minL(a)+minL(b) and thus

(2.3) Λ(ab)≤Λ(a)+Λ(b) .

The *catenary degree* c(a) of an element a∈H is the smallest $N\in N_{0}\cup \{\infty \}$ with the following property: If z,z′∈Z(a) are two factorizations of *a*, then there are factorizations $z=z_{0},z_{1},\ldots ,z_{k}=z\prime$ of *a* such that $d(z_{i-1},z_{i})\leq N$ for all i∈[1,k]. Then

$$c(H)=\sup\{c(a)|a\in H\}\in N_{0}\cup \{\infty \}$$

is the *catenary degree* of *H*. The monoid *H* is factorial if and only if c(H)=0, and if this does not hold, then

(2.4) 2+supΔ(H)≤c(H) by [18,Theorem1.6.3].

**Primary monoids and domains.** Let *H* be a monoid and $m=H\setminus H^{\times }.$ Then, *H* is said to be

- *primary* if $H\neq H^{\times }$ and for all a,b∈m there is an n∈N such that $b^{n}\in aH,$ and
- *strongly primary* if $H\neq H^{\times }$ and for every a∈m, there is an n∈N such that $m^{n}\subseteq aH$ (we denote by M(a) the smallest n∈N having this property).

If *H* is strongly primary, then *H* is a primary BF-monoid ([18, Lemma 2.7.7]). However, primary BF-monoids need not be strongly primary (Example 3.17.2).

Lemma 2.3. [15, Proposition 1] *Let H be a primary monoid. Then* $H^{\times }=H\prime^{\times }\cap H={\tilde{H}}^{\times }\cap H={\hat{H}}^{\times }\cap H.$

Lemma 2.4. Let H be a primary monoid.

*1. If* a∈H *and* x∈q(H)*, then there exists an* N∈N *such that* $a^{N}x\in H$*, so* $a^{n}x\in H$ *for all* n≥N*. Moreover, for n sufficiently large, we have both* $a^{n}x\in H$ *and* $a^{n}\in Hx.$

*2. If H is strongly primary and* x∈q(H)*, then, for* N∈N *sufficiently large,* $m^{N}x\subseteq H$ *and* $m^{N}\subseteq Hx.$

Proof. 1. Let $x=bc^{-1},$ where b,c∈H. We have $Hb\subseteq Hbc^{-1}=Hx.$ Since *H* is primary, *Hb* and so also *Hx* contain a power of *a*. Hence, also $Hx^{-1}$ contains a power of *a*. Thus for *n* sufficiently large we have both $a^{n}x\in H$ and $a^{n}\in Hx.$

2. Use a similar argument as in the previous item. □

If *H* is strongly primary and x∈q(H), we denote by M(x)∈N the smallest n∈N with $m^{n}\subseteq xH$ (see Lemma 2.4.2).

Lemma 2.5. Let H be a primary monoid.

1. $H\prime =\tilde{H}$*. In particular, H is seminormal if and only if H is root-closed.*

*2. If H is seminormal, then* $m\subseteq (H:\hat{H})$*, so* $\hat{H}=(m:m).$

*3. [*14*, Theorem 4]* $\hat{\tilde{H}}$ *is completely integrally closed. Thus the complete integral closure of a seminormal primary monoid is completely integrally closed.*

Proof. 1. By (2.1), we have $H\prime \subseteq \tilde{H}$ whence it remains to verify the reverse inclusion. Let x∈q(H), and k∈N such that $x^{k}\in H.$ By Lemma 2.4.1, there is an N∈N such that ${\left(x^{k}\right)}^{n}x\in H$ for all n≥N. Thus $x^{kn}\in H$ and $x^{kn+1}\in H$ for all n≥N, which implies that $x^{m}\in H$ for all *m* sufficiently large since *kn* and *kn* + 1 are coprime integers (explicitly, $x^{m}\in H$ for $m\geq {\left(kn\right)}^{2}$). Therefore x∈H′.

2. Let $x\in \hat{H},$ thus $dx^{n}\in H$ for some d∈H and all n∈N. Let a∈H. Since *H* is primary, we have $a^{k}d^{-1}\in H$ for some k∈N, so $a^{k}x^{n}\in H$ for all n∈N. Thus, $a^{k}{\left(x^{n}\right)}^{k}\in H$ for all n∈N. Since *H* is root-closed by item 1., we obtain that $ax^{n}\in H$ for all n∈N. Thus, $a\hat{H}\subseteq H,$ so $m\hat{H}\subseteq H.$ Since m⊆n by Lemma 2.3, we infer that $m\hat{H}\subseteq m,$ that is, $\hat{H}\subseteq (m:m),$ so $\hat{H}=(m:m).$ Cf. [19, Proposition 4.8]. □

Monoid properties do not always carry over to integral domains. However, the domain *R* is seminormal (completely integrally closed, Mori, Krull, primary, strongly primary, atomic) if and only if its monoid $R^{•}$ has the respective property. We consider, for example, the Mori property. By definition, the domain *R* is Mori if and only if it satisfies the ascending chain condition on integral divisorial ideals. If X⊆R is a subset, then I=(R:(R:X)) is divisorial, and we have $I^{•}=(R^{•}:(R^{•}:X^{•})),$ and $I=(R^{•}:(R^{•}:X^{•}))\cup \{0\},$ where $Y^{•}=Y\setminus \{0\}$ for Y⊆q(R). It follows that the domain *R* is Mori if and only if the monoid $R^{•}$ is Mori.

Note that a domain *R* is primary if and only if *R* is one-dimensional and local ([18, Proposition 2.10.7]). Every primary Mori monoid is strongly primary with *v*-$\text{spec}(H)=\{\emptyset ,H\setminus H^{\times }\}$ whence every one-dimensional local Mori domain is strongly primary ([18, Proposition 2.10.7]. A survey on the ideal theory of Mori domains is given by Barucci in [6]).

All finitely primary monoids (including all numerical monoids) are strongly primary ([18, Section 2.7]). Examples of finitely primary domains which are not Mori can be found in [26, Sections 3 and 4]. Moreover, [26, Examples 4.6 and 4.7] are not multiplicative monoids of domains; Example 4.6 is Mori, while Example 4.7 is not. Puiseux monoids (these are additive submonoids of $\left(Q_{\geq 0},+)\right),$ which are strongly primary, are discussed in [16] and strongly primary monoids stemming from module theory can be found in [4, Theorems 5.1 and 5.^3^].

An example going back to Grams ([2, Example 1.1] or [18, Example 1.1.6]) exhibits an atomic one-dimensional local domain which is not a BF-domain whence it is neither strongly primary nor locally tame (see Definition 3.1 below).

## 3. On the arithmetic of strongly primary monoids and domains

We start with the concept of local tameness as given in [18].

Definition 3.1. Let *H* be an atomic monoid.

1. For an element a∈H, let ω(a) denote the smallest $N\in N_{0}\cup \{\infty \}$ with the following property: If n∈N and $a_{1},\ldots ,a_{n}\in H$ with $a|a_{1}\cdot \ldots \cdot a_{n},$ then there is a subset Ω⊂[1,n] such that

$$|\Omega |\leq N\;\text{and}\;a|\prod_{\lambda \in \Omega }a_{\lambda }\;.$$

We set ω(H)=sup{ω(u)|u∈A(H)}.

2. For an element $u\in A(H_{\text{red}}),$ let t(u) denote the smallest $N\in N_{0}\cup \{\infty \}$ with the following property: If a∈H with Z(a)∩uZ(H)≠∅ and z∈Z(a), then there is a z′∈Z(a)∩uZ(H) such that d(z,z′)≤N.

3. *H* is said to be

*(a) locally tame* if t(u)<∞ for all $u\in A(H_{\text{red}})$ and

*(b)* (*globally*) *tame* if $t(H)=\sup\{t(u)|u\in A(H_{\text{red}})\}<\infty .$

If u∈A(H), we let $t(u)=t(uH^{\times }).$ For a prime element u∈H, we have ω(u)=1 and t(u)=0, thus ω(H)=1 for a factorial monoid. If u∈A(H) is not prime, then ω(u)≤t(u) whence for a nonfactorial monoid we have ω(H)≤t(H). Moreover, *H* is globally tame if and only if ω(H)<∞ [23, Proposition 3.5]. Every Mori monoid satisfies ω(a)<∞ for all a∈H ([21, Theorem 4.2], [10, Proposition 3.3]) but this need not hold true for the local tame degrees t(·). If *H* is an atomic monoid, since supL(a)≤ω(a) for all a∈H ([21, Lemma 3.3]), the finiteness of the ω(a) values (hence, in particular, local tameness) implies that *H* is a BF-monoid.

We continue with a simple reformulation of local tameness which we use in the following. Clearly, for an atom $u\in A(H_{\text{red}}),$ the local tame degree t(u) is the smallest $N\in N_{0}\cup \{\infty \}$ with the following property:

- For every multiple $a\in H_{\text{red}}$ of *u* and any factorization $z=v_{1}\cdot \ldots \cdot v_{n}$ of *a* which does not contain *u*, there is a subproduct which is a multiple of *u*, say $v_{1}\cdot \ldots \cdot v_{m},$ and a refactorization of this subproduct which contains *u*, say $v_{1}\cdot \ldots \cdot v_{m}=uu_{2}\cdot \ldots \cdot u_{\ell }$ such that max{ℓ,m}≤N.

Recall that a monoid is *half-factorial* if all the factorizations of an element in *H* are of the same length, equivalently, if the set of distances Δ(H) is empty.

Proposition 3.2. Let H be a strongly primary monoid.

*1. For every atom* u∈H*, we have* ω(u)≤M(u)*. Thus* ω(u)<∞.

*2. Assume that* Λ(H)<∞*. Then* $\rho_{\Lambda (H)}(H)=\infty$*, so* $\rho_{k}(H)=\infty$ *for all* k≥Λ(H)*. In particular, H is not half-factorial.*

*3. For every atom* u∈H *we have* $t(u)\leq \rho_{\omega (u)}(H)$*. Hence, if* $\rho_{\omega (u)}(H)<\infty$ *for every atom* u∈H*, then H is locally tame.*

Proof. 1. Since *u* divides each product of M(u) nonunits in *H*, we see that N=M(u) satisfies the property in Definition 3.1.1, and since ω(u) is the least *N* satisfying this property, it follows that ω(u)≤M(u).

2. For every n∈N, a product of *n* atoms is also a product of *k* atoms for some positive integer k≤Λ(H). Hence, $\rho_{\Lambda (H)}(H)\geq \rho_{k}(H)\geq n.$ Thus, $\rho_{\Lambda (H)}(H)=\infty .$

3. Let *a* be an element of *H* that is divisible by an atom *u*. A factorization of *a* has a subproduct that is divisible by *u* and of length k≤ω(u). Hence, every factorization of this subproduct is of length $\leq \rho_{k}(H)\leq \rho_{\omega (u)}(H).$ By the reformulation of Definition 3.1.1, we obtain that $t(u)\leq \rho_{\omega (u)}(H).$ □

A monoid *H* is said to be a

- *valuation monoid* if for all a,b∈H we have a|b or b|a.
- *discrete valuation monoid* if $H_{\text{red}}≅(N_{0},+).$

Lemma 3.3. *Let H be a monoid with* $m=H\setminus H^{\times }\neq \emptyset .$

1. *H is a primary valuation monoid if and only if H is a completely integrally closed valuation monoid if and only if* $H_{\text{red}}$ *is isomorphic to a monoid of non-negative elements of a nonzero subgroup of* (R,+).
2. *The following conditions are equivalent:*
  - H is a discrete valuation monoid.
  - *H is atomic and* m *is principal.*
  - H is a completely integrally closed strongly primary monoid.
3. *[24, Theorem 16.4 (g)]* *A valuation monoid is discrete if and only if it is atomic if and only if it is strongly primary.*

Proof. For 1. and for the equivalence of the first two items of 2. we refer to [24, Theorems 15.5 and 16.^4^] and to [14, Section 3]. To complete the proof of 2., we note that (a)⇒(c), so it is enough to prove the implication (c)⇒(b). Thus, it remains to show that m is principal. Let u∈A(H). Then, $m^{M(u)}\subseteq uH.$ If M(u)=1, then m=uH and we are done. Assume that M(u)>1. Then, $m^{M(u)}\subseteq uH,$ whence $u^{-1}m^{M(u)}\subseteq m$ and $u^{-1}m^{M(u)-1}\subseteq (m:m).$ Since *H* is completely integrally closed, we have (m:m)⊆H and thus $m^{M(u)-1}\subseteq uH,$ contradicting the minimality of M(u). □

Lemma 3.4. *Let* (H,m) *be a primary monoid and* $n=\hat{H}\setminus {\hat{H}}^{\times }.$

*1. If every element of* n *has a power lying in* m*, then* $\hat{H}$ *is primary.*

*2. If* $(H:\hat{H})\neq \emptyset$*, then* $\hat{H}$ *is primary if and only if every element of* n *has a power lying in* m.

Proof. 1. Let x,y∈n. There exists an n∈N such that $x^{n}=m\in m.$ Since *H* is primary, by Lemma 2.4.1., there exists a k∈N such that $m^{k}\in yH.$ Thus, $x^{nk}\in yH,$ implying that $\hat{H}$ is primary.

2. Assume that $\hat{H}$ is primary. Let $c\in m(H:\hat{H})$ and x∈n. As $\hat{H}$ is primary, and m⊆n by Lemma 2.3, we obtain that for some integer n∈N we have $x^{n}\in c\hat{H}\subseteq m.$ We complete the proof using item 1. □

Lemma 3.5. *Let* (H,m) *be a strongly primary monoid and* $n=\hat{H}\setminus {\hat{H}}^{\times }.$

*1. If* x∈q(H)*, then* Λ(H∖xH)<M(x)<∞.

*2. We have* Λ(H)<∞ *if and only if there is a* c∈m *with* $\Lambda (\{c^{m}|m\in N_{0}\})<\infty .$

*3. If* Λ(H)=∞*, then every element of* n *has a power lying in* m*, so* $\hat{H}$ *is primary.*

Proof. 1. If a∈H∖xH, then $a\notin m^{M(x)}\subseteq xH.$ Thus, Λ(H∖xH)<M(x).

2. Let c∈m such that $\Lambda (\{c^{m}|m\in N_{0}\})<\infty .$ Let a∈H. By [18, Lemma 2.7.7.1], we have $a|c^{k}$ for some k∈N, so there is an n≤k in $N_{0}$ such that $a=c^{n}b,$ where b∈H is not divisible by *c*. Now 1. implies that

$$\Lambda (a)\leq \Lambda (c^{n})+\Lambda (b)\leq \Lambda (\{c^{m}|m\in N_{0}\})+M(c)\;.$$

Thus, $\Lambda (H)\leq \Lambda (\{c^{m}|m\in N_{0}\})+M(c),$ and the reverse implication is trivial.

3. Suppose there is an x∈n such that no power of *x* belongs to m, and we will prove that Λ(H)<∞. Let d∈m such that $dx^{n}\in H$ for all n∈N. We choose an element c∈m and assert that Λ(c)<M(d)+M(x), implying that Λ(H)<M(d)+M(x). If c∉dH, then Λ(c)<M(d) by 1. Suppose that c∈dH. Then, there exists an integer k∈N such that $c\in (dx^{k}H\setminus dx^{k+1}H),$ because otherwise we would have $c{\left(1/x\right)}^{n}\in H$ for all n∈N implying that $1/x\in \hat{H}.$ Thus, $c=(dx^{k})b,$ where b∈H∖xH whence Λ(b)<M(x) by 1. If $dx^{k}\in dH,$ then $x^{k}\in H\setminus m,$ so *x* is invertible in *H*, a contradiction. This implies that $dx^{k}\in H\setminus dH$ whence $\Lambda (dx^{k})<M(d)$ by 1. Thus, $\Lambda (c)=\Lambda (dx^{k}b)\leq \Lambda (dx^{k})+\Lambda (b)<M(d)+M(x).$ By Lemma 3.4, $\hat{H}$ is primary. □

The converse of Lemma 3.5.3 is false even for domains (see Example 3.15 below and Lemma 3.4). Let (H,m) be a strongly primary monoid. If $\hat{H}$ is not primary (equivalently, if m is not the unique prime s-ideal of $\hat{H}$), then Λ(H)<∞. In particular, if *R* is a strongly primary domain such that $\hat{R}$ is not local, then Λ(R)<∞.

Lemma 3.6. *Let* (H,m) *be a strongly primary monoid such that* Λ(H)=∞*. Let* $\tilde{m}=\tilde{H}\setminus {\tilde{H}}^{\times }$ *and* $n=\hat{H}\setminus {\hat{H}}^{\times }$*. Then*

1.

$$\tilde{m}=n$$;

2. *For every* x∈n*, we have* $x^{n}\in m$ *for all sufficiently large* n∈N;

3. *If* $(H:\hat{H})\neq \emptyset$*, then* $\hat{H}$ *is a primary valuation monoid.*

Proof. 1. By 2.1, we have $\tilde{H}\subseteq \hat{H},$ so by Lemma 2.3, we infer that $\tilde{m}\subseteq n.$ Since Λ(H)=∞, Lemma 3.5.3 implies that every element of n has a power lying in m, whence $n\subseteq \tilde{H}\setminus {\tilde{H}}^{\times }=\tilde{m}.$

2. Let x∈n. Since Λ(H)=∞, Lemma 3.5.3 implies that there is a k∈N such that $x^{k}\in m.$ Since *H* is primary, there is a $q_{0}\in N$ such that $x^{q_{0}k+r}={\left(x^{k}\right)}^{q_{0}}x^{r}\in m$ for all r∈[0,k−1]. If n∈N with $n\geq q_{0}k,$ then n=qk+r, where $q\geq q_{0}$ and r∈[0,k−1], and $x^{n}=x^{k(q-q_{0})}x^{q_{0}k+r}\in m.$

3. By Lemma 3.4, $\hat{H}$ is primary.

Assume to the contrary, that $\hat{H}$ is not a valuation monoid. Thus, there exists an element x∈q(H) such that $x,x^{-1}\notin \hat{H}.$ If $s\in (H:\hat{H}),$ then $s^{n}x,s^{n}x^{-1}\in H$ for all sufficiently large n∈N. Hence, there exists $c\in (H:\hat{H})$ such that $cx,cx^{-1}\in H.$ For each k∈N, let $n_{k}\in N$ be the smallest integer such that $c^{n_{k}}x^{k}\in H,$ and let ${\hat{n}}_{k}\in N$ be the smallest integer such that $c^{{\hat{n}}_{k}}x^{k}\in \hat{H}.$ Thus, by definition, $1\leq {\hat{n}}_{k}\leq n_{k}\leq k,{\hat{n}}_{k+1}\leq {\hat{n}}_{k}+1.$ Also, $c^{n_{k}}x^{k}$ is not divisible by *c* in *H*: For *n_k_* = 1, this holds since $x^{k}\notin H,$ and for $n_{k}>1,$ this follows from the minimality of *n_k_*. Since $c\hat{H}\subseteq H,$ we obtain that $n_{k}\leq {\hat{n}}_{k}+1,$ whence ${\hat{n}}_{k}\leq n_{k}\leq {\hat{n}}_{k}+1.$ As $\hat{H}$ is root-closed by Lemma 2.2.1, we obtain by Lemma 2.1.1 (applied to $\hat{H}$) that the sequence ${\hat{n}}_{k}$ is increasing. Thus, we infer that $|n_{k+1}-n_{k}|\leq |{\hat{n}}_{k+2}-{\hat{n}}_{k}|\leq 2.$ Since $(H:\hat{H})\neq \emptyset ,$ Lemma 2.2 (items 2 and 3) implies that $\hat{H}$ is completely integrally closed and that, for every $m\in N,c^{m}x^{k}\in \hat{H}$ for just finitely many *k*’s. This implies that $\underset{k\to \infty }{\lim}{\hat{n}}_{k}=\infty$ whence $\underset{k\to \infty }{\lim}n_{k}=\infty .$

Proceeding in the same way with the element $x^{-1}$ as with the element *x*, we obtain a sequence $n\prime_{k}$ having all the properties of the sequence ${\left(n_{k}\right)}_{k\geq 1}$ with respect to the element $x^{-1}.$ Then for all k∈N, the element

$$c^{n_{k}+n\prime_{k}}=(c^{n_{k}}x^{k})(c^{n\prime_{k}}x^{-k})$$

is a product of two elements not divisible by *c* in *H*.

Let n∈N and let k∈N be maximal such that $n_{k}+n\prime_{k}\leq n.$ Then $n_{k}+n\prime_{k}\leq n\leq n_{k+1}+n\prime_{k+1}\leq n_{k}+n\prime_{k}+4.$ This implies that

$$c^{n}=c^{n_{k}+n\prime_{k}}c^{f}=(c^{n_{k}}x^{k})(c^{n\prime_{k}}x^{-k})c^{f}\;\text{for}\;\text{some}\;\;f\in [0,4],$$

whence $\Lambda (c^{n})\leq \Lambda (c)+\Lambda (c)+\Lambda (c^{f}).$ Thus, Lemma 3.5.2 implies that Λ(H)<∞, a contradiction. □

Theorem 3.7. *Let (R, M) be a strongly primary domain. If* $\left(R:\hat{R})=(0\right)$*, then* Λ(R)<∞.

Proof. Assume to the contrary that Λ(R)=∞. Let $n=\hat{R}\setminus {\left(\hat{R}\right)}^{\times }.$ Choose a nonzero element c∈M. Let *n* be a positive integer. Since $\left(R:\hat{R})=(0\right),$ we infer that (cn−1n)R^⊆R, whence there exists an element x∈n such that $c^{n-1}x\notin R.$

Since Λ(R)=∞, Lemma 3.6.2 implies that $x^{i}\in M$ (equivalently, $x^{i}\in R$) for all sufficiently large integers i∈N. Let i∈N be maximal such that $c^{n-1}x^{i}\notin R.$ Since *R* is primary, there exists a maximal non-negative integer *k* such that $c^{n-1}c^{k}x^{i}\notin M.$ Set $y=c^{k}x^{i},$ so $c^{n-1}y\notin R.$ We have $c^{n}y^{j}\in M$ for all j∈N and $c^{n-1}y^{j}\in M$ for all *j* > 1 since $c^{n-1}x^{j}\in m$ for all *j* >* i*. We have $y^{e}\in M$ for some positive integer *e*. Hence

$$(1-y)(1+y+\cdots +y^{e-1})=1-y^{e}\in R^{\times }.$$

Thus

$$c^{n}(1-y)\in R,\;\;\text{and}\;\frac{c^{n}}{1-y}=c^{n}\frac{1+y+\cdots +y^{e-1}}{1-y^{e}}\in R.$$

We see that $c^{n}(1-y)$ and $\frac{c^{n}}{1-y}$ are not divisible by *c* in *R*. Thus, $c^{2n}$ is a product of two elements that are not divisible by *c* in *R*:

$$c^{2n}=(c^{n}(1-y))\;(\frac{c^{n}}{1-y}).$$

Hence, $\Lambda (c^{2n})<M(c)+M(c).$ By Lemma 3.5.2 applied to *c*^2^ replacing *c*, we conclude that Λ(R)<∞, a contradiction. □

Theorem 3.7 is false for monoids by Example 3.17.1 below.

Theorem 3.8.

- *Let* (H,m) *be a strongly primary monoid, and let* $f=(H:\hat{H})$*. Then the first two conditions below are equivalent, and each of the first four conditions implies its successor. Moreover, if* f≠∅*, then all nine conditions are equivalent.*
  1. H is globally tame.
  2. $\underset{u\in A(H)}{\cap }uH\neq \emptyset .$
  3. ρ(H)<∞.
  4. $\rho_{k}(H)<\infty$ *for all* k∈N.
  5. Λ(H)=∞.
  6. $\hat{H}$ *is a primary valuation monoid.*
  7. $\hat{H}$ *is a valuation monoid.*
  8. $fm\subseteq \underset{u\in A(H)}{\cap }u\hat{H}.$
  9. $fm^{2}\subseteq \underset{u\in A(H)}{\cap }uH.$
- *Let (R, M) be a strongly primary domain, and* $f=(R:\hat{R})$*. All the following nine conditions are equivalent.*
  1. R is globally tame.
  2. $\underset{u\in A(H)}{\cap }uH\neq \emptyset .$
  3. ρ(R)<∞.
  4. $\rho_{k}(R)<\infty$ *for all* k∈N.
  5. Λ(R)=∞.
  6. $\hat{R}$ *is a primary valuation domain and* f≠(0).
  7. $\hat{R}$ *is a valuation domain and* f≠(0).
  8. $(0)\neq fm\subseteq \underset{u\in A(R)}{\cap }u\hat{R}.$
  9. $(0)\neq fm^{2}\subseteq \underset{u\in A(R)}{\cap }uR.$

Proof. (a)

1. (1)⇒(2)

By assumption ω(H)<∞. Let c∈m. Then for every a∈A(H), there is an $n_{a}\in N$ such that $a|c^{n_{a}}$ whence $a|c^{\omega (H)}.$ This implies that $c^{\omega (H)}\in \underset{u\in A(H)}{\cap }uH.$

(2)⇒(1) Since *H* is strongly primary, we have $m^{k}\subseteq \underset{u\in A(H)}{\cap }uH$ for some positive integer *k*. Since for every atom *u*, we have ω(u)≤k, it follows that ω(H)<∞, so that *H* is globally tame.

Thus, the first two conditions are equivalent.

(1)⇒(3) See [18, Theorem 1.6.6].

(3)⇒(4) See Equation (2.2).

(4)⇒(5) This follows from Proposition 3.2.2.

Now assume that f≠(0).

(5)⇒(6) See Lemma 3.6.3.

(6)⇒(7) Obvious.

(7)⇒(8) Let *u* be an atom in *H*. Since u∉fm and fm is an *s*-ideal of the valuation monoid $\hat{H},$ it follows that $fm\subseteq u\hat{H}.$ Thus, the assertion follows.

(8)⇒(9)

Let *u* be an atom in *H*. Since $fm\subseteq u\hat{H},$ we infer that $f^{2}m\subseteq uf\hat{H}\subseteq uH.$ The assertion follows.

(9)⇒(2) Obvious.

(b) By Theorem 3.7, condition (5) implies that f≠(0). Thus, each of the first five conditions implies that f≠(0). Obviously, each of the other four conditions implies that f≠(0). By item a., all the nine conditions are equivalent.

Theorem 3.9. 1. *Let H be a strongly primary monoid.*

- *If* Λ(H)<∞*, then H is locally tame, but not globally tame.*
- *If* Λ(H)=∞ *and* $(H:\hat{H})\neq \emptyset$*, then H is globally tame.*
- *H is locally tame if either* Λ(H)<∞*, or* $(H:\hat{H})\neq \emptyset .$

*2. Let R be a strongly primary domain.*

- R is locally tame.
- *R is globally tame if and only if* Λ(R)=∞.

Proof.  

1.

(a) Let $u\in A(H_{\text{red}}),$ *a* be a multiple of *u*, and let $a=v_{1}\ldots v_{n}$ be a product of atoms. There exists a subproduct of $v_{1}\ldots v_{n}$ of length ≤ω(u) that is divisible by *u*. This subproduct has a refactorization of the form *ub* where *b* is a product of at most Λ(H) atoms. Hence, t(u)≤max{ω(u),Λ(H)+1}<∞, so *H* is locally tame.

The monoid *H* is not globally tame by the implication (1)→(5) of Theorem 3.8.

(b) If $(\hat{H}:H)\neq \emptyset ,$ then all nine conditions of Theorem 3.8 (a) are equivalent. In particular, if Λ(H)=∞ (condition (5)), then *H* is globally tame (condition (1)).

(c)This follows immediately from the previous two items.

2. By Theorem 3.7, if $\left(R:\hat{R})=(0\right),$ then Λ(R)<∞. Hence, item 2. (for domains) follows from item 1.(for monoids).

In the next corollary, we answer in the positive Problem 38 in [7].

Corollary 3.10. *A one-dimensional local Mori domain R is locally tame. Moreover, R is globally tame if and only if* Λ(R)=∞.

Proof. A one-dimensional local Mori domain is strongly primary. Thus, the corollary follows from Theorem 3.9.2. □

In the next proposition, we deal with two significant special cases of strongly primary monoids.

Proposition 3.11. *Let* (H,m) *be a strongly primary monoid.*

*1. Let H be seminormal. Then* $(H:\hat{H})⊇m$*, so H is locally tame, and all conditions of Theorem 3.8 (a) are equivalent. If* $\hat{H}$ *is Krull, then H is Mori.*

*2. Let H be Mori with* $(H:\hat{H})\neq \emptyset$*. Then* $\hat{H}$ *is Krull,* ${\hat{H}}_{\text{red}}$ *is finitely generated, and all conditions of Theorem 3.8 (a) are equivalent to* $\hat{H}$ *being a discrete valuation monoid.*

Proof. 1. We have $(H:\hat{H})⊇m$ by Lemma 2.5.2. Thus, *H* is locally tame (Theorem 3.9), and the conditions of Theorem 3.8 (a) are equivalent. If $\hat{H}$ is Krull, then *H* is Mori by [32, Lemma 2.6].

2. Suppose that *H* is Mori and $(H:\hat{H})\neq \emptyset .$ Then, $\hat{H}$ is Krull and ${\hat{H}}_{\text{red}}$ is finitely generated by [18, Theorem 2.7.9]. Since $(H:\hat{H})\neq \emptyset ,$ all conditions of Theorem 3.8 (a) are equivalent to $\hat{H}$ being a valuation monoid (condition (7)). Since Krull monoids are atomic, Lemma 3.3.3 implies that Krull valuation monoids are discrete. □

Proposition 3.12. Let H be a strongly primary monoid that satisfies one of the following two properties.

1. H is not locally tame.

2. Λ(H)=∞ and $(H:\hat{H})=\emptyset .$

Then H is not the multiplicative monoid of a domain.

Proof. For (1) see Theorem 3.9 (b). For (2), see Theorem 3.8 (b). □

For a strongly primary Mori monoid that satisfies both conditions of Proposition 3.12, see Example 3.17.1 below or [16, Proposition 3.12].

Remark 3.13. Let *R* be a strongly primary domain. Then, Theorems 3.7 and 3.^8^ show that either (Λ(R)<∞ and ρ(R)=∞) or (ρ(R)<∞ and Λ(R)=∞). This was proved for one-dimensional local noetherian domains in [27, Corollary 3.7], and it was assumed as an additional abstract property for strongly primary monoids in the study of weakly Krull domains in [28, Corollary 4.11].

We present some examples related to the complete integral closure of a strongly primary domain. Let *R* be a strongly primary domain such that $\left(R:\hat{R})\neq (0\right).$ By Theorem 3.8 (b), if $\hat{R}$ is a valuation domain, then $\hat{R}$ is primary. The converse is false as shown in Example 3.15 below. Moreover, $\hat{R}$ is strongly primary if and only if $\hat{R}$ is a discrete valuation domain by Lemma 3.3.3. In Example 3.16, $\hat{R}$ is a valuation domain, but it is not discrete. On the positive side, $\hat{R}$ is a discrete valuation domain if *R* is Mori by Proposition 3.11.2. Clearly, the domain $\hat{R}$ need not be primary. In particular, if *R* is a one-dimensional local noetherian domain, then $\hat{R},$ which is equal to the integral closure of *R*, is not necessarily a local ring.

For Example 3.15 below, we need Proposition 3.14.

Proposition 3.14.  

1. *Let T be a primary monoid. There exists a strongly primary submonoid* (H,m) *of T such that* $T=\hat{H}$ *and* (H:T)≠∅ *if and only if T is completely integrally closed. Moreover, in this case, the monoid* (H,m) *can be chosen such that* m *is a principal s-ideal of T, whence* T=(m:m) *and* m⊆(H:T)*. The conditions of Theorem 3.8 (a) are satisfied if and only if T is a discrete valuation monoid, and just in this case we may choose H = T.*

2. *Let T be a primary domain. There exists a strongly primary subring (R, M) of T such that* $T=\hat{R}$ *and* (R:T)≠(0) *if and only if T is completely integrally closed. Moreover, in this case, the ring (R, M) can be chosen such that* $T=\hat{R}$ *and the ideal MT of T is generated by two elements. If furthermore, T contains a field, then we may choose R such that M is a principal ideal of T, whence* T=(M:M) *and* M⊆(R:T)*. The conditions of Theorem 3.8 (b) are satisfied if and only if T is a discrete valuation domain, and just in this case we may choose R = T.*

Proof. By Lemma 2.2.2, if *H* is a monoid such that $(H:\hat{H})\neq \emptyset ,$ then $\hat{H}$ is completely integrally closed. Thus, in both statements, we just have to prove the converse. We start with the proof of the second statement.

2. (i) Let $n=T\setminus T^{\times },$ and let *c* be an element of n. Let H=cT∪{1}, and let m=cT. Thus, (H,m) is a submonoid of (T,n). Since *T* is primary, if x∈H, then, $c^{n}\in xT$ for some integer n∈N. Thus, $m^{n+1}c^{n+1}T\subseteq \mathrm{xcT}=cm.$ It follows that *H* is a strongly primary monoid. Also (H:T)⊇m. Hence $T\subseteq \hat{H}.$ Since *T* is completely integrally closed, we obtain that $T=\hat{H}.$ For the last sentence see Lemma 3.3.3.

2. (ii) The domain *T* is local since it is primary. Let *N* be the maximal ideal of *T*. Let *c* be a nonzero element of *N*, and let *F* be the prime field contained in the quotient field of *T*. Set A=(F+cT)∩T=(F∩T)+cT, thus *A* is a subring of *T*. Let P=A∩N=(F∩N)+cT, so *P* is a prime ideal of *A*. Set *R* = *A_P_*, and *M* = *PA_P_*. Thus, (*R*, *M*) is a local subring of (*T*, *N*).

If *T* contains a field (e.g. if *T* has finite characteristic), then $P=cT=PA_{P}=M,$ thus *M* is a principal ideal of *T*. Otherwise, *T* has zero characteristic, and we may identify F=Q. Thus, F∩T is a local subring of Q, that is, a localization of Z at a nonzero prime ideal. Hence, F∩T is a discrete valuation domain. Let *d* be a generator of the maximal ideal of F∩T. Thus, MT=cT+dT.

Let *x* be a nonzero element of *M*, since *T* is primary, and *MT* is a finitely generated ideal of *T*, we have ${\left(MT\right)}^{k}\subseteq \mathrm{cxT}\subseteq xR$ for some positive integer *k*. Hene, $M^{k}\subseteq xR,$ so (*R*, *M*) is strongly primary. Since $\left(0)\neq cT\subseteq (R:\hat{R}\right),$ we infer that $T\subseteq \hat{R}.$ Since *T* is completely integrally closed, we obtain that $T=\hat{R}.$

For the last sentence of item 2. see Lemma 3.3.3.

1. The first statement follows from the second one. Indeed, we may use the monoid $H=R^{•},$ so $\hat{H}=T^{•},$ where *R* and *T* are the domains in 2. □

Example 3.15.

1. There is a strongly primary monoid (H,m) such that $\hat{H}$ is primary completely integrally closed, but not a valuation monoid. Moreover, m is a principal *s*-ideal of $\hat{H},$ so $\hat{H}=(m:m),$ abd $m\subseteq (H:\hat{H}).$ Thus, none of the conditions of Theorem 3.8 (a) holds, in particular Λ(H)<∞.
2. For any field *k*, there is a strongly primary domain (*R*, *M*) containing *k* such that $\hat{R}$ is a primary completely integrally closed domain, but not a valuation domain. Moreover, *M* is a principal ideal of $\hat{R},$ so $\hat{R}=(M:M),$ and $M\subseteq (R:\hat{R}).$ Thus, none of the conditions of Theorem 3.8 (b) holds, in particular Λ(R)<∞.

Proof. First we prove the existence of a domain as in item 2. There exists a primary completely integrally closed domain *T* containing *k* that is not a valuation domain (we refer to [29, 33] and [30], and briefly sketch the idea. Indeed, the field *K* of Puiseux series over the algebraic closure $\bar{k}$ of *k* is algebraically closed and it has a discrete valuation that vanishes on $\bar{k}$ with value group Q. As follows from the cited papers, this fact implies the existence of a primary completely integrally closed domain containing $\bar{k}$). By Proposition 3.14.2., there exists a subring *R* of *T* that is strongly primary and such that $T=\hat{R}$ and *M* is a principal ideal of *T*. Since $\hat{R}$ is not a valuation domain, by Theorem 3.8 (b), none of the nine conditions of this theorem are satisfied, in particular, Λ(R)<∞.

As for item 1., we define $H=R^{•}.$ so $\hat{H}=T^{•},$ where *R* is the domain in item 2. □

Example 3.16. There is a strongly primary domain (*R*, *M*) such that $\hat{R}$ is a primary valuation domain, but not strongly primary and $(R:\hat{R})=M$ is a principal ideal of $\hat{R}.$ Thus, all the conditions of Theorem 3.8 (b) are satisfied, in particular, Λ(R)=∞.

Proof. Let *F* be a field, $A=F[X^{q}|q\;\text{rational},q\geq 1],$ and let *P* be the maximal ideal of *A* generated by the set $\left\{X^{q}|q\;\text{rational}\;,q\geq 1\right\}.$ We set *R* = *A_P_* and *M* = *PR_P_*. Clearly, each nonzero element r∈R is of the form $r=vX^{q},$ where q≥1 is rational and *v* is a unit in *R*, whence $M^{\left\lceil q\right\rceil +1}\subseteq rR.$ Thus, *R* is strongly primary. It is easy to show that $\hat{R}$ is equal to *B_Q_*, where $B=F[X^{q}|q\;\text{rational},q>0]$ and *Q* is the maximal ideal of *B* generated by the set $\left\{X^{q}|q\;\text{rational}\;,q>0\right\}.$ Each nonzero element of $\hat{R}$ is of the form *uX^q^*, where *q* > 0 is rational and *u* is a unit in $\hat{R}.$ Clearly, $\hat{R}$ is a valuation domain and $(R:\hat{R})=M=X\hat{R}.$ □

As mentioned in Section 1, Krull monoids with finite class group and C-monoids are locally tame. Furthermore, finitely generated monoids are locally tame ([18, Theorem 3.1.4]) and hence, the same is true for Cohen-Kaplansky domains and their monoids of invertible ideals ([3, Theorem 4.3]). On the other hand, examples of monoids or domains, that are not locally tame, are rare in the literature. Thus, we end this section with a brief overview.

Example 3.17. 1. In contrast to Theorems 3.7 and 3.^8^ (b), there is a strongly primary monoid *H* with the following properties (see [22, Proposition 3.7 and Example 3.8]):

- *H* is Mori with $(H:\hat{H})=\emptyset$ and $\hat{H}$ is a discrete valuation monoid,
- ρ(H)=Λ(H)=c(H)=∞ and *H* is not locally tame.

Moreover, *H* is a submonoid of a one-dimensional local noetherian domain, although *H* is not the multiplicative monoid of a domain since *H* is not locally tame.

2. For every α∈R∖Q, the additive monoid $H_{\alpha }=\{(x,y)\in N^{2}|y<\alpha x\}\cup \{(0,0)\}\subset (N_{0}^{2},+)$ is a root-closed primary BF-monoid which is neither Mori, nor strongly primary, nor locally tame ([21, Example 4.7]).

3. The additive monoid $H=\{(a,b,c)\in N_{0}^{3}|a>0\text{or}b=c\}\subseteq (N_{0}^{3},+)$ is Mori with catenary degree c(H)=3 (whence, by (2.4), all sets of lengths are arithmetical progressions with difference 1) but it is not locally tame ([25, Example 1]).

4. The domain $R=Q[X^{2},X^{3}]$ is one-dimensional noetherian, $\bar{R}=Q[X]$ is factorial, and $\left(R:\bar{R})=(X^{2}\right).$ Nevertheless, *R* fails to be locally tame ([20, Example 6.11]).

5. Let *H* be a Krull monoid with infinite cyclic class group. Then, *H* is locally tame if and only if its catenary degree is finite if and only if its set of distances is finite ([17, Theorem 4.2]).

6. Let *H* be a Krull monoid with class group *G* such that every class contains a prime divisor. Then, *H* is locally tame if and only if *H* is globally tame if and only if *G* is finite ([21, Theorem 4.4]).

## Funding

This work was supported by the Austrian Science Fund FWF, Project Number P28864-N35.
